# GalliForm, a database of Galliformes occurrence records from the Indo-Malay and Palaearctic, 1800–2008

**DOI:** 10.1038/s41597-020-00690-0

**Published:** 2020-10-13

**Authors:** Elizabeth H. Boakes, Richard A. Fuller, Georgina M. Mace, Changqing Ding, Tzo Tze Ang, Alistair G. Auffret, Natalie E. Clark, Jonathon Dunn, Jennifer Gilbert, Viktor Golovnyuk, Garima Gupta, Ulrike Irlich, Emily Joachim, Kim O’ Connor, Eugene Potapov, Roald Potapov, Judith Schleicher, Sarah Stebbing, Terry Townshend, Philip J. K. McGowan

**Affiliations:** 1 Centre for Biodiversity and Environment Research, University College London, Gower Street, London, WC1E 6BT UK; 2 School of Biological Sciences, University of Queensland, Brisbane, QLD 4072 Australia; 3 College of Biological Sciences and Biotechnology, Beijing Forestry University, Beijing, 100083 China; 4 The Institute of Zoology, Zoological Society of London, Regents Park, London, NW1 4RY UK; 5 Department of Ecology, Swedish University of Agricultural Sciences, Box 7044, 75007 Uppsala, Sweden; 6 National Environment Research Council, Polaris House, North Star Avenue, Swindon, SN2 1EU UK; 7 School of Biology, Newcastle University, Newcastle upon Tyne, NE1 7RU UK; 8 FSBI “Taimyr Reserves”, Talnakhskata str 22, Norilsk, 663305 Russia; 9 Fisheries and Oceans Canada, 200 Kent St, Ottawa, Ontario Canada; 10 Department of Biology, Bryn Athyn College, 2945 College Drive, Bryn Athyn, PA 19009 USA; 11 Zoological Institute, Russian Academy of Sciences, St Petersburg, 199034 Russia; 12 Department of Geography, University of Cambridge, Cambridge, CB2 1QB UK; 13 Birding Beijing, Great Walsingham, Norfolk, NR22 6DR UK

**Keywords:** Biodiversity, Conservation biology, Macroecology, Ecological modelling

## Abstract

Historical as well as current species distribution data are needed to track changes in biodiversity. Species distribution data are found in a variety of sources, each of which has its own distinct bias toward certain taxa, time periods or places. We present GalliForm, a database that comprises 186687 galliform occurrence records linked to 118907 localities in Europe and Asia. Records were derived from museums, peer-reviewed and grey literature, unpublished field notes, diaries and correspondence, banding records, atlas records and online birding trip reports. We describe data collection processes, georeferencing methods and quality-control procedures. This database has underpinned several peer-reviewed studies, investigating spatial and temporal bias in biodiversity data, species’ geographic range changes and local extirpation patterns. In our rapidly changing world, an understanding of long-term change in species’ distributions is key to predicting future impacts of threatening processes such as land use change, over-exploitation of species and climate change. This database, its historical aspect in particular, provides a valuable source of information for further studies in macroecology and biodiversity conservation.

## Abstract (summary)

**Table:** 

Measurement(s)	geographic location • Species • Occupancy
Technology Type(s)	georeferencing • digital curation
Sample Characteristic - Organism	Galliformes sp.
Sample Characteristic - Location	Palearctic Region • Indomalayan Region

Machine-accessible metadata file describing the reported data: 10.6084/m9.figshare.12886931

## Background & Summary

Gathering primary biodiversity data is necessary to improve our knowledge of the ecology and conservation status of species. International commitments such as the Convention on Biological Diversity^1^ call for a halt to biodiversity loss and therefore require data to measure biodiversity change. Recent trends in changes in population sizes or geographical ranges can be used to track progress toward biodiversity targets but longer-term trends are needed if we are to put the status of present-day biota into a proper historical context^2,3^. Similarly, if we are to understand the impacts of climate and land use change on species distributions, historical data are required. Ideally, this biodiversity information must be comprehensive, covering common species as well as threatened, and areas of lower biodiversity as well as hotspots.

Our knowledge of species’ distributions is extremely coarse compared to most other environmental variables^4^. Analyses of species’ geographical ranges often rely on predictions of where a species might occur. Predictions might be gleaned from expert opinion (e.g. https://birdsoftheworld.org/bow/home) (and in some instances may be influenced by historical data), the extent of suitable habitat^5^, gridded survey data^6^ or point occurrences^7^. Prominent conservation datasets such as the Living Planet Index^8^ and IUCN’s species distribution maps (https://www.iucnredlist.org/resources/spatial-data-download) are regularly used to assess rates of biodiversity loss but these data sources do not extend back beyond around 1970. Longer-term trends can reveal major shifts in abundance and composition of biological communities, information that should be considered when setting conservation targets^9^.

While aggregated population trends or extent of occurrence maps are useful conservation tools, primary data allow us to investigate biodiversity loss in far greater detail. For example, if species’ ranges are punctuated with local extinction events we might overlook or underestimate species’ declines because we lack the precision to measure them^10^. Additionally, data summaries may be at coarser resolutions than the original data or missing attributes attached to the original record. Freely available primary data allow new questions to be investigated, for which data summaries might not be suitable.

The avian order Galliformes has relatively high quality historical distribution data. This is in part due to their economic and cultural value and their attraction for collectors and ornithologists^11^. Almost all species are non-migratory, making delimitation of their current and historical ranges more tractable. In recent times they have received much conservation attention through being one of the most threatened avian orders – over 25% of species are threatened (www.iucnredlist.org) and many local extinctions have been reported^12^ (http://datazone.birdlife.org/home). Galliformes are subject to a variety of threats including habitat loss, hunting, and agricultural intensification and disturbance (http://datazone.birdlife.org/home). The order exhibits a wide range of ecological characteristics and life history traits, and occurs in a diversity of habitats, meaning that the Galliformes lend themselves well to macroecological studies^13^.

**Fig. 1:**
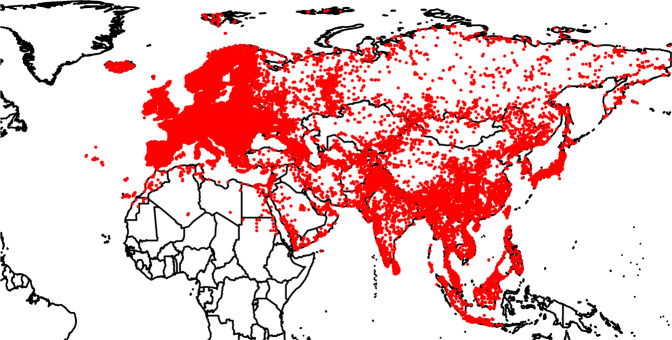
The spatial distribution of those records in GalliForm that contain sufficient information to be georeferenced to an accuracy of 30 minutes. The records of *Lagopus lagopus* and *Lagopus muta* from North America are omitted.

**Fig. 2:**
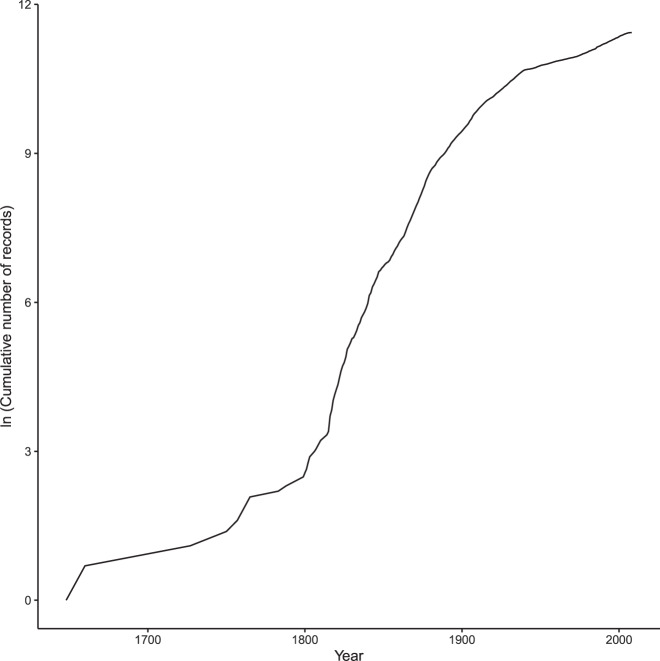
The cumulative number of occurrence records through time. The number of occurrence records has been converted to a natural logarithmic scale.

Here we present GalliForm^14^, a database of 186687 occurrence records covering the 130 species of the avian order Galliformes that occur in the Palaearctic and Indo-Malay biogeographic realms (see Fig. 1 for spatial distribution of records). Records cover the period 1648 to 2008 although 95% of records date from 1877 onwards. Records increase markedly though time (Fig. 2). Records were collected from museums, peer-reviewed and grey literature, bird atlases, banding records and birding trip report websites (see^15^ for spatial biases within sources). Where possible, data were informally refereed by local experts who, if necessary, supplemented the data with their personal records. Each data source was found to have a distinct set of spatial, temporal and taxonomic biases^15^. Combining biodiversity data from a variety of primary sources helps to minimise data bias.

The GalliForm dataset^14^ is an extremely valuable resource for ecological and conservation studies. Occurrence data underpin species distribution modelling but geographic ranges are changing rapidly due to the diverse impacts caused by human activities. Historical occurrence data, coupled with climate and land-use data, may improve our understanding of populations’ responses to climate change, land-use change and hunting. The species occurrence data described here have been used to assess the completeness of geographic range size estimates^16^, to investigate patterns of range collapse with respect to distance to range edge^17^ and to assess species extirpations outside Protected Areas^12^. Nine publications^10,12,15–21^ have so far arisen from this database but many avenues remain to be explored.

## Methods

These methods are an expanded version of those in our related work, Boakes *et al*.^15^.

The database was compiled over the period 2005–2008. Data collection equates to around 1500 person-days and data were gathered by a team of 21 people. Between them, team members were fluent in English, French, German, Mandarin, Russian, Spanish and Swedish. These languages were extremely helpful in transcribing museum specimen labels and in translating publications. However, the majority of publications were in English and we acknowledge that the database will be biased toward records published in English-language publications.

Our study focuses on the 130 galliform species that occur within the Palaearctic and Indo-Malay biogeographic realms^22^ (see Online-only Table 1). We have additionally included records of the Imperial Pheasant (*Lophura imperialis*) although it is now recognised that this is a hybrid and not a species. The geographic range of two of the species in the database, the Red Grouse (*Lagopus lagopus)* and the Rock Ptarmigan (*Lagopus muta*), extends to North America. North American data was often included in the information which museums sent us and in these instances we entered those records into the database since we thought they might be of use to researchers studying these species. However, it should be noted that we did not search exhaustively for records of these species in North America, we have merely included those that we came across.

We attempted to gather all species distribution data that could be accessed from five different sources; museum collections, literature records, banding (ringing) data, ornithological atlases and birdwatchers’ trip report websites. For each data source, exhaustive and systematic search strategies were adopted.

### Museum collections

Using web-based searches and Roselaar^23^, 377 natural history collections were identified. We found contact details for 338 of these collections and requested by email or letter a list of the Galliformes in their holdings along with collection localities and dates. Non-respondents were recontacted. 135 museums were able to share data with us (see Online-only Table 2). Museum records were obtained through publicly available online databases e.g. ORNIS, electronic or paper catalogues sent to us by the museums or by visiting the museums and transcribing data directly from specimens or card catalogues. Almost half of the museums we contacted did not respond despite at least one follow-up enquiry, and there was substantial variation in the amount and format of data contributed by those that did reply. Altogether, over 50% of the records came from just six museums (Natural History Museum, London; Zoological Institute of the Russian Academy of Sciences, St Petersburg; Zoological Museum of Lomonosov Moscow State University; Field Museum of Natural History, Chicago; American Museum of Natural History, New York; National Museum of Natural History, Leiden), a single museum (the Natural History Museum, London) contributing nearly 20% of the museum records that could be georeferenced and dated^15^. Following databasing and/or georeferencing, records were returned to larger collections and to those who had requested the data.

### Literature

Data from the literature were added to those previously collected by McGowan^24^. Entire series of key English-language international and regional ornithological journals such as *Ibis, Bird Conservation International, Journal of the Bombay Natural History Society*, and *Kukila* were scanned for relevant information, availability allowing. We began at the library of the Zoological Society of London and followed up missing journal issues at the BirdLife International library, Cambridge UK; the British Library, London, UK; the Edward Grey Institute, University of Oxford, UK. Relevant Chinese literature was also scanned. Additionally, data were obtained from regional reports, personal diaries, letters, newsletters etc stored in the archives of BirdLife International, Cambridge, UK; the World Pheasant Association, Newcastle, UK; the Edward Grey Institute, University of Oxford, UK. Several of the species/regional experts we consulted also contributed their personal records which were recorded in the database as ‘personal communications’. As far as it were possible, records were classed as primary or secondary data within the ‘dynamicProperties’ field of GalliForm^14^. It is important to note that some primary records or museum specimens will be duplicated within the database in the secondary data.

### Banding records

Eighty-three ornithological banding groups were identified using web-based searches and were contacted via email. Thirty of these groups replied and only seven were able to provide us with data (see Table 1). The majority of galliform species tend not to be banded due to their large body sizes and spurs. Additionally, many of the banding groups kept their records on paper and were not able to send them to us. Nevertheless, we were able to access and georeference 15,152 banding records.

**Table 1:** The ringing groups that shared data with GalliForm.

Ringing group
EURING
Zagreb Ringing Scheme
Hungarian Bird Ringing Centre
Finnish Museum of Natural History, Ringing Centre
Beringungszentrale Hiddensee
Coturnix ringing records, Italy
National Parks Board, Singapore (Ringing Centre)

### Ornithological atlases

We digitised location data from 20 ornithological atlases (see Table 2). Data from several other atlases were not used since the range of dates for the records was wider than 20 years.

**Table 2:** The atlases that were digitised to be included in GalliForm.

Atlas	Year	Editors
The EBCC atlas of European breeding birds: their distribution and abundance^6^	1997	Hagemeijer, E.J.M. & Blair, M.J.
The atlas of breeding birds in Britain and Ireland^30^	1976	Sharrock, J.T.R.
The new atlas of breeding birds in Britain and Ireland^31^	1993	Gibbons, D.W.
Atlas of breeding birds of the West Midlands^32^	1970	Lord, J., Munns, D.J.
Atlas of the breeding birds of Andorra^33^	2002	Alamany, O., Auclair, R., Bertrand, A.
Atlas des oiseaux nicheurs de Belgique^34^	1988	Devilliers, P., Roggeman, W., Tricot, J., Del Marmol, P., Kerwijn, C., Jacob, J-P., Anselin, A.
Atlas of breeding birds in Luxembourg^35^	1987	Melchior, E.
Atlas van de Nederlandse Broedvogels 1973–1977^36^	1979	Teixeira, R.M.
Atlas van de Nederlandse Broedvogels 1978–1983^37^	1987	
Atlas das aves que nidificam em Portugal Continental^38^	1989	Rufino, R.
Atlante degli uccelli nidificanti e svernanti in Toscana^39^	1997	Florenzano, G.T., Arcamone, E., Baccetti, N., Meschini, E., Sposimo, P.
Atlas Hnizdniho Rozsireni Ptaku V CSSR^40^	1987	Stastny, K., Randik, A., Hudec, K.
Birds of Moscow city and the Moscow region^41^	2006	Kalyakin, M.V., Voltzit, O.V.
Eesti Linnuatlas^42^	1993	Renno, O.
Latvian breeding bird atlas^43^	1989	Priednieks, J., Strazds, M, Strazds, A. and Petrins, A.
Zimski ornitoloski atlas Slovenije^44^	1993	Sovinc, A.
Breeding bird atlas of Oman^45^	1998	Eriksen, J.
An interim atlas of the breeding birds of Arabia^46^	1995	Jennings, M.C.
Distribution atlas of Sudan’s birds with notes on habitat and status^47^	1987	Nikolaus, G.
Atlas of wintering birds of Japan^48^	2004	

### Trip report website data

We used the two trip report websites that were popular with birders during the data recording period (2005–2008), www.travellingbirder.com and www.birdtours.co.uk. At that time, eBird (probably the most relevant current online source today) did not cover the majority of the countries within our study region, and our intention with the deposition of this dataset is to focus on pre-eBird data that are more difficult and time consuming to access. We extracted data from all trip reports of birdwatching visits to European, Asian and North African countries. Care was taken to enter reports that featured on both websites once only.

### Criteria for data inclusion

To be included in the database, records had to meet the following criteria:

1. The record identified the species of the bird concerned.
2. The record contained either a verbal description of the locality at which the bird concerned was observed or the co-ordinates at which the bird was observed.

Records of captive birds were excluded. Records relating to non-native occurrences were included but were flagged in the ‘establishmentMeans’ field as “introduced”.

### Data entry

GalliForm^14^ was originally compiled in the programme Microsoft Access 2003. To maximise uniformity in data entry, all data recorders were given thorough and consistent training and each was provided with a set of database guidelines. An Access Database form was created to standardise data entry and to enable multiple members of the team to collect data simultaneously.

Each entry in GalliForm^14^ corresponds to a single record of a single species recorded in a specific location. The data fields of GalliForm^14^ are described in Online-only Table 3. The taxonomy used has been updated to be consistent with the BirdLife International 2019 taxonomy (datazone.birdlife.org). All information was entered exactly as it was described in the data source, with as much information extracted as possible. Multiple records from different sources which recorded the same information were still included in the interest of completeness. The only exception to this is the trip report data in which we did not enter identical records which occurred on both the Travelling Birder and Bird Tours websites.

The source of the data, i.e. literature, museum, atlas, ringing or website trip report is recorded in the ‘dynamicProperties’ field under the code “dataSource”. For literature data, (where known) the nature of the record, i.e. primary or secondary, is recorded under the code “datatype”.

Taxonomy has of course changed considerably over time. To allow for this we recorded the taxonomy as it was described in the data source in the ‘originalNameUsage’ field. The current taxonomy was then selected from a look-up table. If at the time of data entry, the data compiler was unsure which species the synonym referred to, the species was tagged as “unknown” and the species was designated at a later date following further research on the synonym.

Identical localities can also be described in multiple ways. We recorded the locality as it was given in the data source in the ‘verbatimLocality’ field. If the ‘verbatimLocality’ clearly tallied with a locality already within the database, the record was linked to that locality in order to increase georeferencing efficiency.

It was rare for a source to record absence of evidence, i.e. a survey for a species at a particular locality which failed to find that species. However, in the few cases where we did come across such records, the locality and date of the survey were recorded and “absent” was recorded in the ‘occurrenceStatus’ field.

Each record refers to an independent observation. For museum and ringing records, this means a single individual. For literature, atlas or trip report records this may refer to a group of birds observed in one particular locality, on one particular day. If given, the number of total individuals is recorded in the ‘individualCount’ field. The number of males and females is recorded in the ‘sex’ field and the number of juveniles and adults in the ‘lifeStage’ field. If the ‘lifeStage’ field is blank, it is reasonable to assume the individual(s) is an adult.

Occasionally, additional information about the observation might be included in the data source, for example the habitat the bird was observed in or whether the bird was common or rare in that locality. These data are recorded in the ‘habitat’ and ‘organismQuantity’ fields, respectively. Any additional information which did not fit within the structure of the database was recorded in the ‘occurrenceRemarks’ field, along with any notes found on museum labels.

For the purposes of data deposition, the database was converted to a tab-delimited CSV file with all fields following Darwin Core format. A full summary of these fields is given in Online-only Table 3.

### Georeferencing

Locality descriptions were converted to geographic co-ordinates using a wide range of atlases and gazetteers, co-ordinates generally only being assigned if accurate to one degree (although in the majority of cases the locations were accurate to within 30 minutes, Table 3). We would initially search for a locality within the gazetteers available to us at the time. If the locality was not listed within those gazetteers we would search for the locality using atlases. Since this fieldwork was conducted, MaNIS standards have become widely used for studies of this kind, but these weren’t fully developed at the time of data collection^25^. Named places, e.g. towns or counties, were georeferenced using their geographic centre and georeferencing uncertainty measured from the centre to the edge of the named place. Often localities were given simply as the name of a river, mountain or Protected Area. In these instances we used the midpoint of the river between source and mouth (uncertainty measured as distance from midpoint to source/mouth), the summit of the mountain (uncertainty measured as distance from summit to approximate mountain foot) and the rough centre of the Protected Area (uncertainty measured as distance from centre to Protected Area edge). If a particular locality description matched two or more places their midpoint was taken (uncertainty measured as distance from midpoint to place). Offsets from localities (e.g. “50 km N of Kuala Lumpur”; “8 miles along the road from Sheffield to Chesterfield”) were measured using a digital atlas (uncertainty was approximated at the georeferencer’s discretion in these instances, usually between 3 and 10 arc-minutes, depending on the vagueness of the offset.) For georeferencing done ‘in house’, the gazeteer/atlas used was recorded.

**Table 3:** Georeference and date completeness of the records.

Record Class	No. records	No. georeferenced to within 2 minutes	No. georeferenced to within 10 minutes	No. georeferenced to within 30 minutes	No. dated to within one year	No. dated to within 10 years	No. georeferenced to within 30 minutes and dated to within one year
Event	186687	57173 (31%)	58773 (32%)	152930 (82%)	91973 (49%)	165312 (89%)	65913 (35%)
Locality	118907	26282 (22%)	26755 (23%)	109651 (92%)	N/A	N/A	N/A

When possible, localities we could not georeference ourselves were sent to regional experts.

92% of our localities are georeferenced to an accuracy of 30 minutes, corresponding to 82% of occurrence records (see Table 3).

We had less success at georeferencing museum records than literature records^15^, due in part to difficulties in reading hand-writing on specimen labels. Older records were also harder to georeference, presumably due to changes in place names over time, and to some early ornithologists failing to document the collection locality. As might be expected, localities from countries that do not use the Roman alphabet were also harder to georeference.

Some records were excluded from the database based on their locality: records which we thought were trading localities, notably Malacca in Malaysia and Leadenhall Market in the UK; records from captive specimens, e.g. zoological gardens.

### Dating

49% of records are dated to within an accuracy of one year. Where possible, we assigned date ranges to undated records. For example, if the name of the collector was given on a museum specimen and we knew when that collector was active in that region, we assigned a date range covering that period. There remain undated records which could perhaps be dated in this way. Undated literature records were designated as occurring before their publication date. We were able to date 89% of records to within 10 years.

## Data Records

A relational database structure was created in Microsoft Access to organise and store the species occurrence records with their spatial dependencies and data sources and to keep track of synonyms. For the purposes of publication, this database was converted to a tab-delimited CSV file that followed the Darwin Core format.

We provide a dataset for Galliformes occurrences within the Palaearctic and Indo-Malay realms at species level. These data, obtained and curated as explained above, are available from the Global Biodiversity Information Facility (https://doi.org/10.15468/9825yw). Online-only Table 3 lists and describes the fields of GalliForm^14^.

**Fig. 3:**
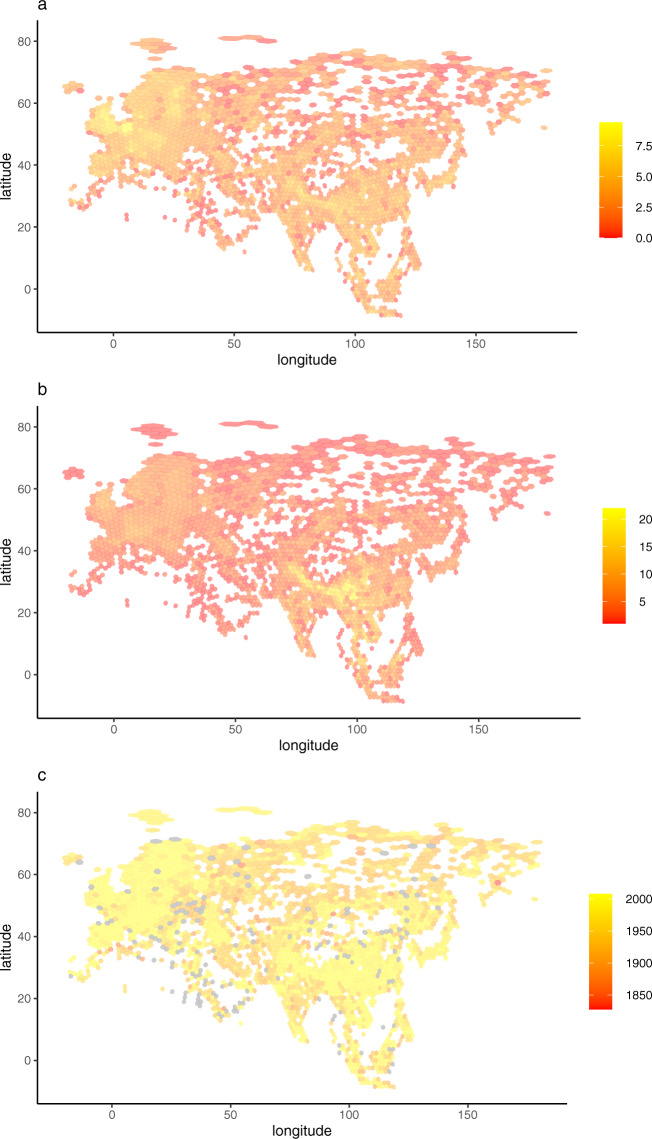
The spatial distribution of the records, coloured coded by (**a**) the natural logarithm of the number of records within each cell, (**b**) the number of species within each cell and (**c**) the most recent year of record within each cell (cells which do not contain any dated records are shaded light grey). Cells are equal area and represent approximately 23,322 km^2^. Cells were drawn using the dgGridR package^28^ in R^29^.

**Fig. 4:**
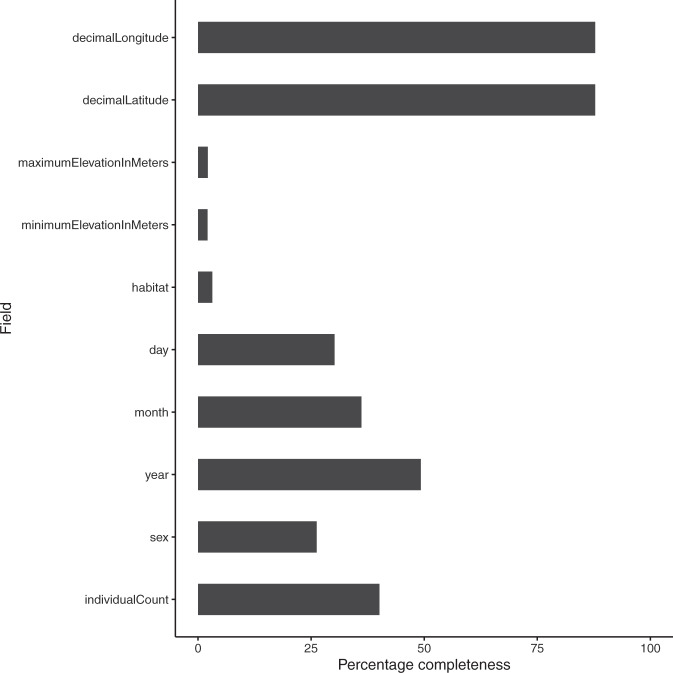
Percentage of data completeness of selected fields of GalliForm. Field descriptions are given in Online-only Table 3.

The following figures and tables summarise the dataset. Figure 1 shows the spatial distribution of records; Fig. 2 shows the accumulation of records through time; Fig. 3 shows the spatial distribution of the number of records, species richness and the most recent year of record; Fig. 4 shows the completeness of selected data fields. Table 1 lists the ringing groups which were able to share data with us; Table 2 lists the atlases that we digitised; Table 3 details the completeness of records which are georeferenced and/or dated to within 1 year. Online-only Table 1 details the number of records per species and the time span these records cover; Online-only Table 2 lists the museums which were able to share data with us; Online-only Table 3 describes the Field Names of GalliForm^14^.

## Technical Validation

Georeferenced data were subject to the following checks:

1. That each data point was in the country that its locality described.
2. That each data point was within reasonable distance of the species’ known historical range.
3. That each data point that identifiably came from a protected area listed in the World Database of Protected Areas (https://www.protectedplanet.net/) was indeed within that protected area.

Finally, data were sent to experts on regions/species for informal ‘refereeing’ to highlight dubious or missing data. We were able to referee approximately one third of the records in this way.

## Usage Notes

The dataset described here can be used to investigate the spatial and temporal patterns of Galliformes distributions at multiple scales and resolutions. The dataset was first used to examine bias in different sources of biodiversity data^15^. It has also been used to investigate predictors of range change^18^, to examine the effects of missing data on estimates of biodiversity metrics^10^, to assess the completeness of geographic range estimates^16^, to investigate the position of local extinctions with respect to species’ range edges^17^, to explore the optimisation of Protected Area networks^20^, to examine the local extirpation of species outside Protected Areas^12^ and to model the potential distributions of highly threatened species^19,21^. There remains much scope for this database to inform further biodiversity or conservation related studies, for example, investigations of geographic range change or predictors of extinction risk.

The data presented here do need to be interpreted carefully with respect to data bias and to missing data. Biodiversity data may be biased in a variety of ways, for example geographically, towards particular ecosystems or towards more charismatic species e.g.^26,27^. Additionally, these data biases may change over time. Although our database is based on a systematic and thorough search of all the data available to us from all regions covered, the data are still likely to be biased because there will have been intrinsic biases in the available data sources. For example, in this database, central India is under-represented in terms of recent research locales and it is hard to disentangle whether this is due to a lower number of ecologists focussing their studies there or if it is a justified skew as a result of biodiversity loss in this area. More recent records also show a bias toward threatened species and Protected Areas^15^. There are very few records of species absence although of course absence may be inferred if there are many records of other species in a particular locality. For a more detailed discussion of bias and missing data see Boakes *et al*.^15^ and Boakes *et al*.^10^.

## Online-only Tables

**Online-only Table 1:** Summary of records of each species in GalliForm. Species taxonomy is that accepted by the IUCN and BirdLife.

Species	Common name	Number of records	Number of localities in which recorded	Year of earliest record	Year of most recent record
*Alectoris barbara*	Barbary Partridge	715	328	1821	2007
*Alectoris chukar*	Chukar	2992	1702	1830	2007
*Alectoris graeca*	Rock Partridge	1058	713	1820	2006
*Alectoris magna*	Przewalski’s Partridge	112	76	1872	2007
*Alectoris melanocephala*	Arabian Chukar	193	149	1852	2006
*Alectoris philbyi*	Philby’s Rock Partridge	75	32	1845	1998
*Alectoris rufa*	Red-legged Partridge	5683	4320	1817	2007
*Ammoperdix griseogularis*	See-see Partridge	694	381	1836	2006
*Ammoperdix heyi*	Sand Partridge	534	264	1820	2006
*Arborophila ardens*	Hainan Hill-partridge	108	54	1891	2005
*Arborophila atrogularis*	White-cheeked Hill-partridge	254	133	1803	2007
*Arborophila brunneopectus*	Brown-breasted Hill-partridge	479	243	1873	2006
*Arborophila cambodiana*	Cambodian Hill-partridge	66	36	1927	2006
*Arborophila campbelli*	Malaysian Hill-partridge	38	22	1907	2000
*Arborophila charltonii*	Chestnut-necklaced Hill-partridge	85	48	1896	1994
*Arborophila chloropus*	Scaly-breasted Hill-partridge	477	253	1873	2007
*Arborophila crudigularis*	Taiwan Hill-partridge	157	74	1864	2007
*Arborophila davidi*	Orange-necked Hill-partridge	42	28	1925	2006
*Arborophila gingica*	Collared Hill-partridge	116	86	1899	2004
*Arborophila graydoni*	Sabah Hill-partridge	102	52	1833	2001
*Arborophila hyperythra*	Bornean Hill-partridge	117	43	1887	2001
*Arborophila javanica*	Javan Hill-partridge	234	56	1826	2002
*Arborophila mandelii*	Chestnut-breasted Hill-partridge	104	73	1876	2007
*Arborophila orientalis*	Grey-breasted Hill-partridge	65	14	1896	1989
*Arborophila rolli*	Tan-breasted Hill-partridge	27	15	1898	2000
*Arborophila rubrirostsris*	Red-billed Hill-partridge	107	33	1878	2001
*Arborophila rufipectus*	Sichuan Hill-partridge	133	82	1921	2007
*Arborophila rufogularis*	Rufous-throated Hill-partridge	946	433	1847	2007
*Arborophila sumatrana*	Sumatran Hill-partridge	31	18	1826	1939
*Arborophila tonkinensis*	Tonkin Hill-partridge	49	16	1925	2006
*Arborophila torqueola*	Necklaced Hill-partridge	712	350	1841	2007
*Argusianus argus*	Great Argus	706	342	1836	2004
*Bambusicola fytchii*	Mountain Bamboo-partridge	447	215	1876	2007
*Bambusicola sonorivox*	Taiwan Bamboo-partridge	232	85	1861	2007
*Bambusicola thoracicus*	Chinese Bamboo-partridge	1010	867	1861	2007
*Bonasa bonasia*	Hazel Grouse	6004	4226	1815	2007
*Bonasa sewerzowi*	Severtzov’s Grouse	300	194	1873	2007
*Caloperdix oculeus*	Ferruginous Partridge	201	113	1860	2004
*Catreus wallichii*	Cheer Pheasant	913	436	1820	2007
*Chrysolophus amherstiae*	Lady Amherst’s Pheasant	414	260	1869	2007
*Chrysolophus pictus*	Golden Pheasant	494	357	1863	2007
*Coturnix coromandelica*	Black-breasted Quail	494	261	1829	2006
*Coturnix coturnix*	Common Quail	14805	9962	1810	2007
*Coturnix japonica*	Japanese Quail	1022	531	1837	2007
*Crossoptilon auritum*	Blue Eared-pheasant	251	139	1869	2007
*Crossoptilon crossoptilon*	White Eared-pheasant	342	196	1890	2007
*Crossoptilon harmani*	Tibetan Eared-pheasant	160	96	1880	2007
*Crossoptilon mantchuricum*	Brown Eared-pheasant	274	164	1866	2005
*Falcipennis falcipennis*	Siberian Spruce Grouse	162	116	1840	1994
*Francolinus francolinus*	Black Francolin	1724	803	1819	2007
*Francolinus gularis*	Swamp Partridge	443	234	1846	2007
*Francolinus pictus*	Painted Francolin	277	161	1845	2001
*Francolinus pintadeanus*	Chinese Francolin	802	521	1788	2007
*Francolinus pondicerianus*	Grey Francolin	962	576	1829	2007
*Galloperdix bicalcarata*	Sri Lanka Spurfowl	168	61	1865	2006
*Galloperdix lunulata*	Painted Spurfowl	173	91	1832	2007
*Galloperdix spadicea*	Red Spurfowl	389	197	1814	2007
*Gallus gallus*	Red Junglefowl	2706	1524	1801	2007
*Gallus lafayettii*	Sri Lanka Junglefowl	466	168	1827	2006
*Gallus sonneratii*	Grey Junglefowl	435	234	1660	2007
*Gallus varius*	Green Junglefowl	271	118	1820	2004
*Haematortyx sanguiniceps*	Crimson-headed Partridge	102	52	1893	2001
*Ithaginis cruentus*	Blood Pheasant	1456	680	1845	2007
*Lagopus lagopus*	Red Grouse	11545	6625	1750	2007
*Lagopus muta*	Rock Ptarmigan	5280	2366	1800	2006
*Lerwa lerwa*	Snow Partridge	387	218	1822	2007
*Lophophorus impejanus*	Himalayan Monal	1055	631	1648	2007
*Lophophorus lhuysii*	Chinese Monal	210	114	1869	2006
*Lophophorus sclateri*	Sclater’s Monal	294	171	1879	2007
*Lophura bulweri*	Bulwer’s Pheasant	202	140	1874	2001
*Lophura diardi*	Siamese Fireback	342	179	1819	2007
*Lophura edwardsi*	Edward’s Pheasant	108	59	1922	2000
*Lophura erythrophthalma*	Malay Crestless Fireback	121	68	1818	1998
*Lophura ignita*	Bornean Crested Fireback	321	153	1836	2003
*Lophura imperialis*	Imperial Pheasant	34	26	1923	2000
*Lophura inornata*	Salvadori’s Pheasant	122	50	1878	2005
*Lophura leucomelanos*	Kalij Pheasant	1861	967	1836	2007
*Lophura nycthemera*	Silver Pheasant	1221	704	1841	2007
*Lophura pyronota*	Bornean Crestless Fireback	119	62	1843	1999
*Lophura rufa*	Malay Crested Fireback	251	102	1807	2004
*Lophura swinhoii*	Swinhoe’s Pheasant	232	97	1863	2007
*Lyrurus mlokosiewiczi*	Caucasian Grouse	340	192	1866	2006
*Lyrurus tetrix*	Black Grouse	11915	6829	1819	2007
*Megapodius cumingii*	Philippine Megapode	106	68	1866	2006
*Megapodius nicobariensis*	Nicobar Megapode	116	45	1860	1998
*Melanoperdix niger*	Black Wood Partridge	209	102	1826	1991
*Ophrysia superciliosa*	Himalayan Quail	48	29	1865	1989
*Pavo cristatus*	Common Peafowl	726	484	1851	2007
*Pavo muticus*	Green Peafowl	1065	620	1828	2007
*Perdicula argoondah*	Rock Bush Quail	228	105	1836	2007
*Perdicula asiatica*	Jungle Bush Quail	665	298	1839	2007
*Perdicula erythrorhyncha*	Painted Bush Quail	197	81	1840	2007
*Perdicula manipurensis*	Manipur Bush Quail	80	38	1881	2006
*Perdix dauurica*	Daurian Partridge	883	513	1855	2007
*Perdix hodgsoniae*	Tibetan Partridge	578	321	1850	2007
*Perdix perdix*	Grey Partridge	32425	29877	1727	2007
*Phasianus colchicus*	Common Pheasant	40451	36456	1783	2007
*Phasianus versicolor*	Green Pheasant	197	81	1845	2006
*Polyplectron bicalcaratum*	Grey Peacock-pheasant	733	435	1838	2007
*Polyplectron chalcurum*	Sumatran Peacock-pheasant	129	56	1848	2004
*Polyplectron germaini*	Germain’s Peacock-pheasant	116	74	1880	2007
*Polyplectron inopinatum*	Mountain Peacock-pheasant	73	36	1902	2008
*Polyplectron katsumatae*	Hainan Peacock-pheasant	55	34	1905	2004
*Polyplectron malacense*	Malayan Peacock-pheasant	132	58	1851	2003
*Polyplectron napoleonis*	Palawan Peacock-pheasant	192	59	1831	2005
*Polyplectron schleiermacheri*	Bornean Peacock-pheasant	78	49	1888	2003
*Pucrasia macrolopha*	Koklass Pheasant	1145	681	1825	2007
*Rheinardia ocellata*	Crested Argus	216	101	1886	2003
*Rhizothera dulitensis*	Dulit Partridge	11	7	1894	1902
*Rhizothera longirostris*	Long-billed Wood Partridge	162	100	1818	2003
*Rollulus rouloul*	Crested Partridge	805	329	1806	2004
*Synoicus chinensis*	King Quail	1185	546	1839	2007
*Syrmaticus ellioti*	Elliot’s Pheasant	415	278	1871	2006
*Syrmaticus humiae*	Mrs Hume’s Pheasant	473	244	1840	2004
*Syrmaticus mikado*	Mikado Pheasant	106	56	1897	2007
*Syrmaticus reevesii*	Reeves’ Pheasant	426	249	1839	2002
*Syrmaticus soemmerringii*	Copper Pheasant	478	321	1833	2004
*Tetrao urogalloides*	Black-billed Capercaillie	369	244	1828	2004
*Tetrao urogallus*	Western Capercaillie	5736	3830	1816	2007
*Tetraogallus altaicus*	Altai Snowcock	153	72	1834	2004
*Tetraogallus caspius*	Caspian Snowcock	137	83	1869	2007
*Tetraogallus caucasicus*	Caucasian Snowcock	166	96	1840	1994
*Tetraogallus himalayensis*	Himalayan Snowcock	720	417	1841	2006
*Tetraogallus tibetanus*	Tibetan Snowcock	507	323	1870	2007
*Tetraophasis obscurus*	Verreaux’s Monal Partridge	141	97	1869	2007
*Tetraophasis szechenyii*	Szechenyi’s Monal Partridge	220	133	1892	2007
*Tragopan blythii*	Blyth’s Tragopan	389	187	1838	2007
*Tragopan caboti*	Cabot’s Tragopan	305	144	1868	2004
*Tragopan melanocephalus*	Western Tragopan	766	429	1841	2006
*Tragopan satyra*	Satyr Tragopan	527	298	1845	2007
*Tragopan temminckii*	Temminck’s Tragopan	577	368	1869	2007

**Online-only Table 2:** The museums that shared data with GalliForm.

Museum	Country
Australian National Wildlife Collection, CSIRO, Australia	Australia
Museum Victoria, Melbourne, Australia	Australia
South Australian Museum	Australia
Biologie Zentrum des Oberostereichisches Landesmuseums, Linz, Austria	Austria
Natural History Musuem, Vienna, Austria	Austria
Institut Royal des Sciences Naturelles de Belgique, Belgium	Belgium
Plodiv Natural Science Museum, Bulgaria	Bulgaria
Ruse Natural History Museum, Bulgaria	Bulgaria
Canadian Museum of Nature	Canada
Royal Alberta Museum, Canada	Canada
Beijing Institute of Zoology, China	China
Nature Museum of Sichuan University, China	China
Normal University of Xihuan, China	China
Muzeum J A Komenskeho, Prerov, Czech Republic	Czech Republic
Vlastivedne Muzeum v Olomouci, Czech Republic	Czech Republic
Naturhistoriska Museum, Aarhus, Denmark	Denmark
University of Copenhagen Museum of Zoology, Denmark	Denmark
Chelmsford Museum, Essex, UK	UK
Zooloogia Muuseum, Tartu, Estonia	Estonia
Musee Guimet d’Histoire Naturelle, France	France
Musee Zoologique de l’Universite Louis Pasteur et de la Ville de Strasbourg, France	France
Museum d’Histoire Naturelle de Grenoble, France	France
Museum d’Histoire Naturelle de Bordeaux, France	France
Museum National d’Histoire Naturelle, Paris, France	France
Institut fur Vogelforschung ’Vogelwarte Helgoland’, Wilhelmshaven, Germany	Germany
Museum für Naturkunde Berlin, Germany	Germany
Museum fur Naturkunde, Magdeburg, Germany	Germany
Naturhistoriches Museum Mainz, Germany	Germany
Naturkunde Museum im Ottoneum, Kassel, Germany	Germany
Pfalzmuseum fur Naturkunde, Bad Duerkheim, Germany	Germany
Senckenberg Museum, Forschungsinstitut Senckenberg (FIS), Germany	Germany
Staatliches Museum fur Naturkunde, Karlsruhe, Germany	Germany
Staatliches Museum fur Naturkunde, Stuttgart, Germany	Germany
Uberseemuseum, Bremen, Germany	Germany
Universitaet Halle, Germany	Germany
Westfalisches Museum fur Naturkunde, Munster, Germany	Germany
Zoologischen Sammlung der Universitat Rostock, Germany	Germany
Zoologisches Forschungsinstitut und Museum Alexander Koenig, Germany	Germany
Zoologisches Institut und Zoologisches Museum, Hamburg, Germany	Germany
Zoologisches Museum der Christian-Albrechts Universitat, Germany	Germany
Zoological Museum Amsterdam, Netherlands	Netherlands
Regional Museum of Natural History, India	India
Museum of Zoology, Bogor, Indonesia	Indonesia
National Museum of Ireland	Ireland
Coll. "A. Noro", City of Graglia (Biella), Italy	Italy
Museo Civico de Storia Naturale ’Giacomo Doria’, Genoa, Italy	Italy
Museo Civico di Storia Naturale di Carmagnola, Italy	Italy
Museo di Storia Naturale del Mediterraneo, Livorno, Italy	Italy
Museo di Storia Naturale di Terrasini, Italy	Italy
Museo Ornitologico ’F. Foschi’, Italy	Italy
Museo Regionale di Scienze Naturali, Torino, Italy	Italy
Museo Zoologico de La Specola, Florence, Italy	Italy
Museo Zoologico dell’ Accademia del Fisiocrtici, Italy	Italy
Universita di Pavia, Italy	Italy
Kaunas Zoological Museum, Lithuania	Lithuania
Ulster Museum, Belfast, UK	N Ireland
Fries Natuurmuseum, Leeuwarden, Netherlands	Netherlands
National Museum of Natural History, Leiden, Netherlands	Netherlands
Auckland Museum, New Zealand	New Zealand
Museum of Natural History and Archaeology, Trondheim, Norway	Norway
Universitets Museet I Tromso, Norway	Norway
Zoologisk Museum, Bergen, Norway	Norway
Museum of Natural History, Wroclaw University, Poland	Poland
Zaklad Zoologii Systematycznej I Doswiadczalnej, Poland	Poland
Museo Municipal do Funchal, Portugal	Portugal
Museu Bocage, Lisbon, Portugal	Portugal
Museu de Historia Natural-Zoologia, Porto, Portugal	Portugal
Muzeul ’Tarii Crisurilor’, Oradea, Romania	Romania
Zoological Institute RAS, St Petersburg, Russia	Russia
Zoological Museum of Moscow University (ZMMU), Moscow, Russia	Russia
Zoological Reference Collection, Singapore	Singapore
South African Museum, Cape Town, South Africa	South Africa
Estacion Biologica de Donana, Seville, Spain	Spain
Museo Nacional de Ciencias Naturales, Madrid, Spain	Spain
Ajtte Svensk Fjall- och Samemuseum, Sweden	Sweden
Malmo Museer, Sweden	Sweden
Naturhistoriska Museet, Gothenburg, Sweden	Sweden
Swedish Museum of Natural History, Stockholm, Sweden	Sweden
Zoologisk Museum, Lund, Sweden	Sweden
Musee Zoologie, Lausanne, Switzerland	Switzerland
Museum d’Histoire Naturelle de la Ville de Geneve, Switzerland	Switzerland
Museum d’Histoire Naturelle de Neuchatel, Switzerland	Switzerland
Naturhistorisches Museum Bern, Switzerland	Switzerland
Naturhistorisches Museum, Basel, Switzerland	Switzerland
Zoologisches Museum der Universitat Zurich-Irchel, Switzerland	Switzerland
Booth Museum of Natural History, Brighton, UK	UK
Bristol Museums and Art Gallery Service, UK	UK
Dorman Museum, Middlesbrough, UK	UK
Glasgow Art Gallery and Museum, UK	UK
Great North Museum: Hancock, UK	UK
Leicester City Museums Service, UK	UK
Liverpool Museum, UK	UK
Manchester Museum, University of Manchester, UK	UK
National Museums and Galleries of Wales	UK
Nottinghamshire Biological and Geological Records Centre, UK	UK
Oxford University Museum of Natural History, UK	UK
Royal Albert Memorial Museum and Art Gallery, UK	UK
Saffron Walden Museum, UK	UK
Shropshire County Museum Service, UK	UK
The Herbert Museum, Coventry, UK	UK
The Natural History Museum, London, UK (BMNH)	UK
Tullie House Museum and Art Gallery, Carlisle, UK	UK
British Library National Sound Archive (NSA), UK	UK
University Museum of Zoology Cambridge, UK	UK
Academy of Natural Sciences, Philadelphia, USA	USA
American Museum of Natural History, New York, USA	USA
Bernice P. Bishop Museum, Hawai’i, USA	USA
Borror Laboratory of Bioacoustics, Ohio, USA	USA
Burke Museum of Natural History and Culture, Seattle, USA	USA
California Academy of Sciences, USA	USA
Carnegie Museum of Natural History, Pittsburgh, USA	USA
Cleveland Museum of Natural History, Ohio, USA	USA
Colorado University Museum, USA	USA
Cornell University Museum of Vertebrates, UK	USA
Delaware Museum of Natural History, USA	USA
Denver Museum of Nature and Science, USA	USA
Donald R Dickey Bird and Mammal Collection, UCLA, USA	USA
Florida Museum of Natural History, USA	USA
Humboldt State University Wildlife Museum, USA	USA
Los Angeles County Museum of Natural History, USA	USA
Michigan State University Museum, USA	USA
Museum of Comparative Zoology, Harvard, USA	USA
Museum of Vertebrate Zoology, Berkeley, USA	USA
Museum of Zoology, University of Michigan (UMMZ), USA	USA
New York State Museum, USA	USA
North Carolina State Museum of Natural Science, USA	USA
Sam Noble Museum of Natural History, University of Oklahoma, USA	USA
Santa Barbara Museum of Natural History, USA	USA
Slater Museum of Natural History, WA, USA	USA
Smithsonian National Museum of Natural History, USA	USA
The Bell Museum, Minnesota, USA	USA
The Field Museum, Chicago, USA	USA
University of Nebraska State Museum, USA	USA
Utah Museum of Natural History, University of Utah, USA	USA
Yale Peabody Museum, USA	USA

**Online-only Table 3:** Explanation of the Field Names in GalliForm. All records have the following fields filled: catalogNumber, locality and scientificName.

Field Name	Darwin Core class	Description of contents
institutionCode	Record-level	This name of the institution having custody of the object or information referred to in the record.
basisOfRecord	Record-level	The specific nature of the data record, as defined by the standard labels of the Darwin Core classes, choices being “PreservedSpecimen” or “HumanObservation”.
dynamicProperties	Record-level	The type of data source (coded within the field as “dataSource”) from which the record came, choices being “Literature”; “Museum”; “Atlas”; “Ringing”; “Website Trip Report”. Where known, from the literature were categorised as “primary” or “secondary” (coded as “dataType”).
catalogNumber	Occurrence	A unique number (within GalliForm) for each record.
recordedBy	Occurrence	The name of the person or expedition that collected the specimen.
individualCount	Occurrence	The number of individual birds that the record relates to.
organismQuantity	Occurrence	A qualitative status statement relating to whether the species is common, rare etc in that locality.
sex	Occurrence	The sex of the individual(s) represented by the Occurrence
lifeStage	Occurrence	The life stage of the individuals(s) represented by the Occurrence.
establishmentMeans	Occurrence	Coded as “introduced” if the Occurrence is outside a species’ native range.
occurrenceStatus	Occurrence	A statement about the presence or absence of a taxon at a location
preparations	Occurrence	The medium by which a museum specimen is preserved: Study Skin; Mounted Skin; Sound; Frozen Material; Tissue; Fluid-preserved Carcass; Fluid-preserved Skeleton; Egg; Nest; Skeletal Material; Wings.
associatedReferences	Occurrence	The reference associated with the Occurrence.
otherCatalogNumbers	Occurrence	The catalogue number assigned to a specimen by a museum.
occurrenceRemarks	Occurrence	Any information associated with the record that the data miner perceived as having potential relevance for the user, also any notes given on a museum label.
eventDate	Event	The date or interval when the Event was recorded. 1890–12–2 would mean some time during the day of the 2^nd^ of December, 1890; 1910–11 would mean some time during the month of November 1910, 2002 would mean some time during the year 2002; 1930–1935 would mean some time between the 1^st^ of January 1930 and the 31^st^ of December 1935; /2008 would mean some time before 31^st^ December 2008.
year	Event	The year in which the individual(s) was recorded.
month	Event	The month in which the individual(s) was recorded, numerically coded, i.e. 1 represents January.
day	Event	The day of the month on which the individual(s) was recorded.
habitat	Event	The type of habitat in which the individual was recorded, choices being bush; cultivation; desert; disturbed forest; forest; grassland; meadow; moor; road; rocky; scrubland; taiga; tundra; urban.
eventRemarks	Event	The way the Event was observed: Specimen; Sight Record; Heard Record; Heard and Seen; Second Hand (i.e. the observer was told of the species’ presence by another.)
higherGeography	Location	A list (concatenated and separated) of geographic names less specific than the information captured in the locality term.
country	Location	The name of the country in which the Location occurs. In a few cases, relating to older records, historical major administrative units are referred to e.g. USSR.
locality	Location	The specific description of the Location. The term may contain information modified from the original to correct perceived errors or standardise the description.
verbatimLocality	Location	The original textual description of the locality.
minimumElevationInMeters	Location	The lower limit of the altitude at which the individual(s) was recorded, as measured in metres.
maximumElevationInMeters	Location	The upper limit of the altitude at which the individual(s) was recorded, as measured in metres.
decimalLatitude	Location	The geographic latitude of the Location. Positive values are north of the Equator, negative values are south of it.
decimalLongitude	Location	The geographic longitude of the Location. Positive values are east of the Greenwich Meridian, negative values are west of it.
geodeticDatum	Location	The ellipsoid on which the geographic coordinates given in decimalLatitude and decimalLongitude are based.
coordinateUncertaintyInMeters	Location	The horizontal distance (in metres) from the given decimalLatitude and decimalLongitude describing the smallest circle containing the whole of the Location.
georeferenceProtocol	Location	A reference to the methods used to determine the coordiantes and uncertainties.
georeferenceSources	Location	A list of maps, gazetteers or other sources used to georeferenced the Location.
scientificName	Taxon	The full scientific name, (as given by BirdLife’s taxonomic checklist).
originalNameUsage	Taxon	The taxon name as given by the original data source, e.g. museum label, report.
kingdom	Taxon	The full scientific name of the kingdom in which the Taxon is classified.
phylum	Taxon	The full scientific name of the phylum in which the Taxon is classified.
class	Taxon	The full scientific name of the class in which the Taxon is classified.
order	Taxon	The full scientific name of the order in which the Taxon is classified.
family	Taxon	The full scientific name of the family in which the Taxon is classified.
genus	Taxon	The full scientific name of the genus in which the Taxon is classified.
specificEpithet	Taxon	The name of the species epithet of the scientific name.
vernacularName	Taxon	The vernacular name as given by the original data source, e.g. museum label, report.

## References

[1] Convention on Biological Diversity. *COP 10 Decision X/2 - Strategic Plan for Biodiversity 2011–2020*. (Montreal, Canada, 2010).

[2] Sheppard C (1995). The shifting baseline syndrome. Mar. Poll. Bull..

[3] Willis KJ (2007). How can a knowledge of the past help to conserve the future? Biodiversity conservation and the relevance of long-term ecological studies. Philos. T. R. Soc. B.

[4] Jetz W, McPherson JM, Guralnick RP (2012). Integrating biodiversity distribution knowledge: toward a global map of life. Trends Ecol. Evol..

[5] Rondinini C (2011). Global habitat suitability models of terrestrial mammals. Philos. T. R. Soc. B.

[6] Hagemeijer, W. & Blair, M. *The EBCC Atlas of European Breeding Birds*. (T & AD Poyser, England, 1997).

[7] Sullivan BL (2009). eBird: A citizen-based bird observation network in the biological sciences. Biol. Conserv..

[8] Collen B (2009). Monitoring change in vertebrate abundance: the Living Planet Index. Conserv. Biol..

[9] Balmford A (1999). (Less and less) great expectations. Oryx.

[10] Boakes EH, Fuller RA, McGowan PJK, Mace GM (2016). Uncertainty in identifying local extinctions: the distribution of missing data and its effects on biodiversity measures. Biol. Lett..

[11] McGowan PJK, Garson PJ (2002). The Galliformes are highly threatened: Should we care?. Oryx.

[12] Boakes EH, Fuller RA, McGowan PJK (2019). The extirpation of species outside protected areas. Conserv. Lett..

[13] Madge, S. & McGowan, P. *Pheasants, partridges and grouse: A guide to the pheasants, partridges, quails, grouse, guineafowl, buttonquails and sandgrouse of the world*. (Christopher Helm Publishers Ltd, 2002).

[14] Boakes EH (2020). The Global Biodiversity Information Facility.

[15] Boakes EH (2010). Distorted views of biodiversity: spatial and temporal bias in species occurrence data. PLoS Biol..

[16] Gupta G, Dunn J, Sanderson R, Fuller R, McGowan PJK (2020). A simple method for assessing the completeness of a geographic range size estimate. Glob. Ecol. Conserv..

[17] Boakes EH, Isaac NJB, Fuller RA, Mace GM, McGowan PJK (2018). Examining the relationship between local extinction risk and position in range. Conserv. Biol..

[18] Mace GM, Collen B, Fuller RA, Boakes EH (2010). Population and geographic range dynamics: implications for conservation planning. Philos. T. R. Soc. B.

[19] Dunn JC, Buchanan GM, Cuthbert RJ, Whittingham MJ, McGowan PJK (2015). Mapping the potential distribution of the Critically Endangered Himalayan Quail *Ophrysia superciliosa* using proxy species and species distribution modelling. Bird Conserv. Int..

[20] Dunn JC, Buchanan GM, Stein RW, Whittingham MJ, McGowan PJK (2016). Optimising different types of biodiversity coverage of protected areas with a case study using Himalayan Galliformes. Biol. Conserv..

[21] Grainger MJ, Ngoprasert D, McGowan PJK, Savini T (2019). Informing decisions on an extremely data poor species facing imminent extinction. Oryx.

[22] Olson DM (2001). Terrestrial ecoregions of the world: A new map of life on earth. Bioscience.

[23] Roselaar CS (2003). An inventory of major European bird collections. Bull. Br. Ornithol. Club.

[24] McGowan PJK (1996). Mapping the distribution of Asian partridges and pheasants as a requirement for identifying conservation priorities. Acta Zool. Sinica.

[25] Wieczorek J, Guo Q, Hijmans R (2004). The point-radius method for georeferencing locality descriptions and calculating associated uncertainty. Int. J. Geogr. Inf. Sci..

[26] Isaac NJB, Pocock MJO (2015). Bias and information in biological records. Biol. J. Linn. Soc..

[27] Collen B, Ram M, Zamin T, McRae L (2008). The tropical biodiversity data gap: addressing disparity in global monitoring. Trop. Conserv. Sci..

[28] Barnes, R. dggridR: Discrete Global Grids for R. https://cran.r-project.org/web/packages/dggridR/ (2016).

[29] R Core Team. R: Version 3.5.1. A Language and Environment for Statistical Computing. http://www.R-project.org (2018).

[30] Sharrock, J.T.R. *The Atlas of Breeding Birds in Britain and Ireland* (Poyser, 1977).

[31] Gibbons, D.W. *The New Atlas of Breeding Birds in Britain and Ireland* (Poyser, 1993).

[32] Lord, J. & Munns, D.J. *Atlas of Breeding Birds of the West Midlands* (Collins, 1970).

[33] Alamany, O., Auclair, R. & Bertrand, A. *Atlas of the Breeding Birds of Andorra* (Associasió per a la Defensa de la Natura, Andorra, 2002).

[34] Devilliers, P. *et al*. *Atlas des Oiseaux Nicheurs de Belgique* (Institut Royale des Sciences Naturelles, 1988).

[35] Melchior, E. *Atlas of Breeding Birds in Luxembourg* (Lëtzebuerger Natur- a Vulleschutzliga, 1987).

[36] Teixeira, R.M. *Atlas van de Nederlandse Broedvogels 1973-1977* (Natuurmonumenten 1979).

[37] Atlas van de Nederlandse Broedvogels 1978-1983 (Sovon, 1987).

[38] Rufino, R. *Atlas das Aves que Nidificam em Portugal Continental* (ICNB/Assírio & Alvin 1989).

[39] Florenzano, G.T., Arcamone, E., Baccetti, N., Meschini, E. & Sposimo, P. *Atlante Degli Uccelli Nidificanti e Svernanti in Toscana* (CentrOrnitologicoToscano, 1997).

[40] Stastny, K., Randik, A. & Hudec, K. *Atlas Hnizdniho Rozsireni Ptaku V CSSR* (Academia, 1987).

[41] Kalyakin, M.V. & Voltzit, O.V. *Birds of Moscow City and the Moscow Region* (Pensoft, 2006).

[42] Renno, O. *Eesti Linnuatlas* (Light, 1993).

[43] Priednieks, J., Strazds, M, Strazds, A. & Petrins, A. Latvian Breeding Bird Atlas (Riga Zinatne, 1989).

[44] Sovinc, A. *Zimski Ornitoloski Atlas Slovenije* (Tehniška založba Slovenije, 1994).

[45] Eriksen, J. *Breeding bird atlas of Oman* (Oman Bird Records Committee, 1998).

[46] Jennings, M.C. *An Interim Atlas of the Breeding Birds of Arabia* (National Commission for Wildlife Conseravtion, 1995).

[47] Nikolaus, G. *Distribution Atlas of Sudan*’*s Birds with Notes on Habitat and Status* (Zoologisches Forschungsmuseum Alexander Koenig, 1987).

[48] Atlas of Wintering Birds of Japan (Ministry of Environment, 2004).

